# Myocarditis Induced by Immunotherapy in Metastatic Melanoma—Review of Literature and Current Guidelines

**DOI:** 10.3390/jcm11175182

**Published:** 2022-09-01

**Authors:** Anna M. Czarnecka, Marcin Kleibert, Iga Płachta, Paweł Rogala, Michał Wągrodzki, Przemysław Leszek, Piotr Rutkowski

**Affiliations:** 1 Department of Soft Tissue, One Sarcoma and Melanoma, Maria Sklodowska-Curie National Research Institute of Oncology, 02-781 Warsaw, Poland; 2 Faculty of Medicine, Medical University of Warsaw, 02-091 Warsaw, Poland; 3 Department of Pathology and Laboratory Diagnostics, Maria Sklodowska-Curie National Research Institute of Oncology, 02-781 Warsaw, Poland; 4 Department of Heart Failure and Transplantology, National Institute of Cardiology, 04-628 Warsaw, Poland

**Keywords:** Immune checkpoint inhibitor, adverse event, immunotherapy, melanoma, nivolumab, myocarditis, cardiotoxicity, guidelines

## Abstract

Immunotherapy is a widely used treatment modality in oncology. Immune checkpoint inhibitors, as a part of immunotherapy, caused a revolution in oncology, especially in melanoma therapy, due to the significant prolongation of patients’ overall survival. These drugs act by activation of inhibited immune responses of T lymphocytes against cancer cells. The mechanism responsible for the therapy’s high efficacy is also involved in immune tolerance of the patient’s own tissues. The administration of ICI therapy to a patient can cause severe immune reactions against non-neoplastic cells. Among them, cardiotoxicity seems most important due to the high mortality rate. In this article, we present the history of a 79 year-old patient diagnosed with melanoma who died due to myocarditis induced by ICI therapy, despite the fast administration of recommended immunosuppressive therapy, as an illustration of possible adverse events of ICI. Additionally, we summarize the mechanism, risk factors, biomarkers, and clinical data from currently published guidelines and studies about ICI-related myocarditis. The fast recognition of this fatal adverse effect of therapy may accelerate the rapid introduction of treatment and improve patients’ outcomes.

## 1. Introduction

Over the past decades, significant progress in cancer therapy has been observed. The shift from standard chemotherapy to targeted treatment, and recently to immunotherapy has taken place. The number of patients eligible for this modern treatment is growing rapidly with a simultaneous increase in the number of adverse events (AE) related to immunotherapy. Most organs have been reported to be the targets of immune-related toxicity induced by immune checkpoint inhibitors (ICI) used, e.g., in melanoma treatment [1]. Unfortunately, biomarkers defining populations at risk are not well defined at this point in time, but PD-1/PD-1L pathway proteins are known to be involved in AEs [2]. However, Weidhass et al. showed that germline (microRNA-based biomarker) predicts grade 2 and higher irAEs (immune-related AEs) to anti-PD1/PDL1 therapy regardless of the type of cancer [3].

**Figure 1:**
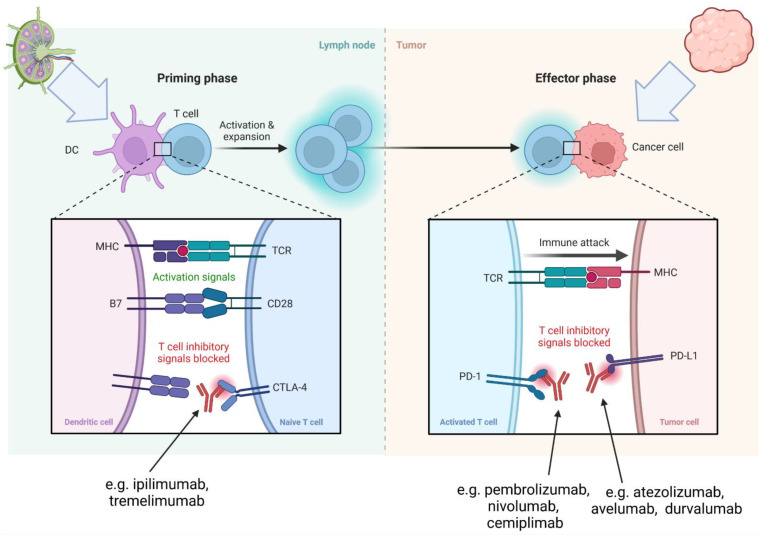
Mechanism of blockade of CTLA-4 or PD-1 signaling in tumor immunotherapy and examples of drugs used in melanoma therapy. CTLA-cytotoxic T cell antigen, PD-programmed cell death protein, MHC-major histocompatibility complex, TCR-T-cell receptor, CD-cluster of differentiation DC-dendritic cell.

Melanoma immunotherapy acts by disinhibition of T-cell function (Figure 1). It can lead to overactivity of the immune system, which results in irAEs. In 2018, a meta-analysis that showed the mortality rate among patients treated with ICI was published. It was reported that 0.36% of those treated with anti–PD-1, 0.38% of those treated with anti–PD-L1, 1.08% of those treated with anti–CTLA-4, and 1.23% of those treated with combined anti–PD-1/ anti–PD-L1 and CTLA-4 died due to complications of the treatment. The type of fatal irAE observed varied by regimen. It can manifest as e.g., colitis, pneumonitis, hepatitis, neurotoxicity, or myocarditis [4].

Cardiotoxicity is an infrequent irAE during melanoma treatment. Among cardiac-related irAEs left ventricular dysfunction, heart failure, myocarditis, myocardial fibrosis, takotsubo syndrome, QT prolongation, arrhythmias, heart block, cardiac arrest, acute coronary syndrome, hypertension, and thromboembolism were reported [5,6,7,8]. The first specific case report of myocarditis during treatment with a PD-1/PD-L1 inhibitor was published in 2014 [9]. Tadokoro et al. (2016) reported probably the first biopsy-proven case of acute lymphocytic myocarditis that occurred after the administration of nivolumab [10]. As was mentioned, cardiac-related complications are not a common example of irAE. According to the safety databases of Bristol-Myers Squibb Corporate, the incidence of myocarditis was first reported to be 0.09% in patients treated with nivolumab, ipilimumab, or both. Patients who received combination therapy with both antibodies had more severe and frequent myocarditis than those who received nivolumab alone (0.27% versus 0.06%) [11]. Two retrospective studies showed a higher prevalence of cardiac events in patients treated with ICIs; 1.0% and 1.14%, respectively [5,12]. However, it often presents with a rapid course, with almost 50% of patients experiencing a major adverse cardiovascular event, which results in death or progression to end-stage dilated cardiomyopathy (rate: 12–50%) [12,13,14,15,16]. The increasing number of cases of myocarditis as an adverse reaction is continuously reported. In the Danish study, the 1-year risk of peri- or myocarditis was 1.8%, thus suggesting that the risk may be higher than previously estimated [17]. The median time to onset was reported to be 17 to 39 days following treatment; however, in an analysis by Matzen et al., the median time to onset of symptoms after initiation of ICI was 16 days, but with a wide variation of 1 to 196 days, whereas Qin et al. in their institutional report showed that the time to irAE ranged from 1.5 to 54 weeks [16,18,19]. Jain et al. reported a wide spectrum of cardiovascular irAEs after ICI therapy with absolute incidence rates of stroke (4.6%), heart failure (3.5%), atrial fibrillation (2.1%), conduction disorders (1.5%), myocardial infarction (0.9%), myocarditis (0.05%), vasculitis (0.05%), and pericarditis (0.2%) [20].

Despite the growing number of cases, pathogenesis is still not known. In our article, we describe the case of fulminant lethal myocarditis related to nivolumab therapy along with PD-1/PD-L1 analysis in the heart. Additionally, we present the current knowledge regarding the pathogenesis, biomarkers, clinical course, and treatment of this rare adverse event during therapy.

## 2. Clinical Background-Case Report

A 79-year-old patient, after resection of pT4b melanoma of the left foot two years previously (weight: 96 kg, height: 164 cm, BSA: 2.09 m^2^, BMI: 35.7 $\frac{\mathrm{kg}}{m^{2}}$), was referred to the tertiary oncology center due to the presence of a nodular lesion of 37 × 31 mm in the 10th segment of the left lung and a 9 mm nodule in the 2nd segment of the right lung in CT. Additionally, the 3 mm lesion was found in the liver, and no metastatic changes were detected in CNS. The patient had arterial hypertension and permanent atrial fibrillation treated according to guidelines by a general practitioner. After excluding the BRAF mutation, he was referred to immunotherapy and started treatment with nivolumab at a constant dose of 240 mg every three weeks. The patient received three courses of treatment within 1.5 months without any complications. However, some changes in laboratory results worsened within this period (Table 1). On admission to the 4th treatment course, he reported a well-being deterioration, according to the family report, it had lasted for 6 days—without general practitioner intervention. He also reported severe headaches, weakness, loss of appetite, and shortness of breath. In physical examination, no significant abnormalities were observed.

An urgent CT scan was performed which excluded pulmonary embolism, stroke, pleural effusion, and CNS metastases. Due to the exacerbation of symptoms and chest pain (very likely nonanginal), an ECG was performed which showed the presence of atrial fibrillation (persistent), without apparent signs of myocardial ischemia. The cardiac biomarkers presented a moderate increase in troponin T: 1.300 ng/mL (<0.014), CK-MB: 90.78 ng/mL (<6.22) and significant in NT-pro-BNP: 2396.0 pg/mL (<125). Echocardiography revealed normal size and slightly reduced contractility of the left ventricle with basal inferior wall hypokinesis and global EF 55–60%. Right ventricle size and contractility were within normal ranges. A six hour follow-up evaluation proved a stable troponin level of 1.030 ng/mL.

A further MRI and endomyocardial biopsy (EMB) were planned; however, due to the patient’s condition, limited access to MRI /EMB and autoimmune reaction related to immunotherapy the treatment was started without further delay. The patient’s condition was worsening gradually and in the evening, he developed delusions. The patient received steroid therapy (prednisolone 1 mg/kg) and acetylsalicylic acid (75 mg). Despite the rapid start of steroid therapy, the patient died within 21 h of admission.

**Figure 2:**
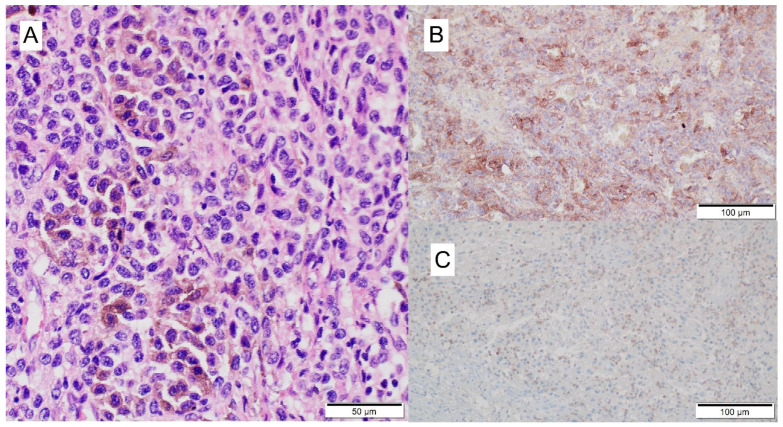
Histopathological presentation of primary lesion—malignant melanoma: Tumor composed of epithelioid melanocytes (**A**), which show PD-L1 expression (**B**); whereas lymphoid cells in the stroma present with PD-1 expression (**C**).

**Figure 3:**
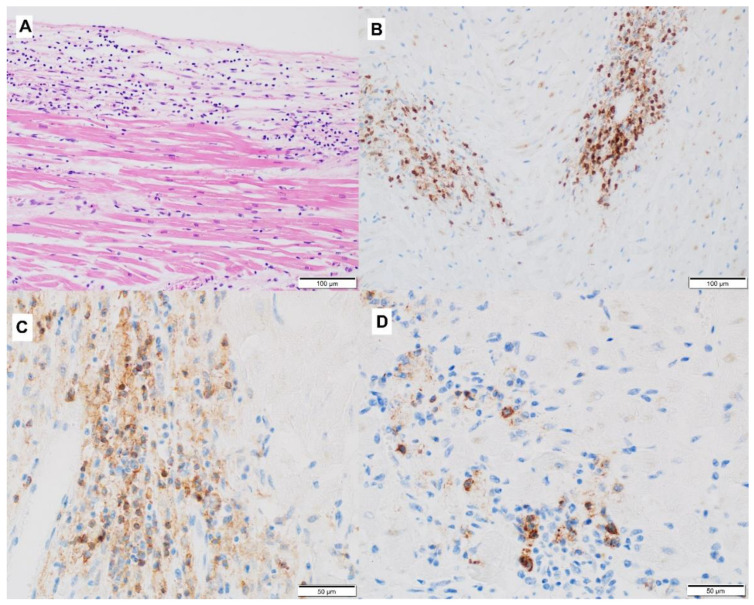
Histologic presentation of cardiotoxicity during nivolumab: H&E stain of the myocardium shows patchy lymphocytic infiltrates associated with myocarditis and myocyte damage (**A**); immunochemistry stain for CD3 (**B**), CD4 (**C**), and CD8 (**D**) highlights in brown T cells within the inflammatory infiltrate.

The autopsy report stated left foot melanoma recurrence, with metastases to the left inguinal lymph node and both lungs (apT4bN1bM1) (Figure 2). The direct cause of death was lymphocytic myocarditis (according to Dallas criteria) from T cells (CD3 (+), CD4 (+), CD8 (+), PD-1 (+), CD20 (−)) (Figure 3).

## 3. Review and Discussion

The first case series of ICI-related cardiotoxicities was a US and German study by Heinzerling et al. (2016), including autoimmune myocarditis, cardiomyopathy, heart failure, cardiac fibrosis, and cardiac arrest [21]. Moslehi et al. described 101 cases reported in VigiBase (http://www.vigiaccess.org/, accessed on 20 August 2022, the World Health Organization (WHO) database of individual safety case reports, whereas Salem et al. identified 122 cases, also reported in VigiBase [13,14]. Matzen et al. identified 87 cases of ICI-induced myocarditis, and 39 among them were melanoma patients [16]. Rahouma et al. performed a meta-analysis of 11 anti-PD/PD-L1 immunotherapy randomized clinical trials (seven melanoma RCTs, three non-small-cell lung cancer RCTs, and one prostate cancer,) including five studies comparing mono-immunotherapy to chemotherapy, which showed that cardiotoxicity was statistically insignificant (RR: 1.15; 95% CI: 0.73–1.80; *p* = 0.55) regardless the treatment regimen; either chemotherapy or dual immunotherapy [22]. All the ICIs can be responsible for development of myocarditis. In a retrospective study among 752 patients by Voskens et al. (2013), one case of ipilimumab-induced myocardial fibrosis was reported [7]. In a phase III trial of adjuvant ipilimumab after complete resection of high-risk stage III melanoma (EORTC 18071), one patient in the ipilimumab group died because of myocarditis. It was probably the first reported death due to ICI-induced myocarditis [23]. Also, a few more cases of fulminant myocarditis were reported, and most of them were fatal [11,24,25,26]. Pericarditis and pericardial effusion were also mentioned. Apical ballooning and cardiomyopathy (a Takotsubo-like syndrome) or pericarditis with pericardial effusion were also reported [27,28]. Scard et al. (2021), reported a case of myocardial infarction (MI) in a patient who previously underwent MI and was treated with triple coronary bypass implantation [18]. The first case of autoimmune myocarditis as a side effect of pembrolizumab therapy was reported by Läubli et al. (2015), completely regressing after high-dose corticosteroids [29]. The rate of adverse cardiovascular events is reported to be low but when it occurs the severity is very high. Mahmood et al. presented that among patients who developed ICI-associated myocarditis, in nearly one-half of cases incidence of grade 4 or 5 for cardiovascular adverse events was noted, graded using the Common Toxicity Criteria for Adverse Events (version 4.0) [12]. By contrast, 4% of cases of pneumonitis after anti-PD-1 or anti-PD-L1 treatment, were grade 4 or 5 [30]. Hofmann et al. presented that the incidence of hepatitis during anti-PD-1 therapy was 2.2% and only 18.2% of patients developed grade 4; no grade 5 was reported. Diarrhea and colitis were only in grades 1–3 [31]. In a research study by Dearden et al., all 2 myocarditis cases were grade 5, and no other treatment-related mortality was reported [32]. There is no evidence that cardiac toxicity correlates with the ICI’s dose [33]. Interestingly, targeted therapies used in melanoma treatment may also induce cardiotoxicity. BRAF/MEK inhibitors may cause reduction in left ventricular ejection fraction (5–11%), hypertension (11–30%) or QT interval prolongation (0–5%) [34]. The summary of the most important information about cases, which are mentioned above, is provided in supplementary data (Table S1 in Supplementary Materials).

### 3.1. Diagnosis of Myocarditis

Myocarditis is an inflammatory disease of cardiac muscle that might occur as a result of infections, predominantly viral, exposure to drugs, or immune system activation [35,36]. Its diagnosis is established using histological, immunological and immunohistochemical criteria [35,37]. For patients who do not undergo EMB or have nondiagnostic findings on EMB, myocarditis cannot be definitely diagnosed. However, in those cases a diagnosis of clinically suspected myocarditis can be made if diagnostic criteria are met. Diagnosis of myocarditis according to a 2013 position statement of the European Society of Cardiology Working Group on Myocardial and Pericardial Diseases is shown in Table 2.

Myocarditis is often classified in terms of duration of symptoms. Acute myocarditis has been defined as a condition with symptoms of heart failure developing over three months or less, while chronic myocarditis has been defined as developing over >3 months [39]. Fulminant myocarditis is also differentiated as a subtype, it has a distinct onset, degree of hemodynamic compromise but has a generally better prognosis than (sub)acute lymphocytic myocarditis symptoms [35,36].

### 3.2. Mechanism of ICI-Related Myocarditis, Biomarkers and Histopathology

The exact mechanism of cardiac IRAEs remains poorly understood; however, it is likely related to the direct inhibition of PD-1 and CTLA-4. The PD-1/PD-L1 pathway seems fundamental for the immune homeostasis within the myocardium and the cardiac protection from T-lymphocytes. Deregulated immune cells were found to mislabel surface structures such as cardiolipin as antigens, leading to subsequent targeting of normal cardiomyocytes or other cells expressing these antigens. Therefore, a mechanism of action for the ICIs resembles cardiovascular complications of patients with autoimmune diseases, including systemic lupus erythematosus [21,40].

Based on early animal models, it was demonstrated that after CTLA-4 inhibition or PD-1 deletion, autoimmune myocarditis may develop [41]. PD-1 is known to protect against tissue inflammation and murine cardiomyocytes damage [42]. It regulates a critical checkpoint for autoimmune myocarditis and cardiomyocyte damage [41].

Moreover, PD-L2 deficiency has been described to predispose to exacerbation of myocarditis in mice [43]. The damage to the gene encoding PD-1 in mice caused dilated cardiomyopathy [44]. Knock-out of the PD-L1/PD-L2 genes or treatment with anti-PD-L1 antibodies were shown to transform transient myocarditis into a lethal form of the disease [45]. These observations were confirmed in the cell culture of human cardiomyocytes. The blockage of PD-1 by nivolumab caused infiltration of the myocardium with T lymphocytes and increased expression of inflammatory genes [46].

Another co-inhibitory molecule is a CTLA-4 which binds to CD80 (also known as B7.1) or CD86 (also known as B7.2), with a ten-fold higher affinity than CD28 (co-stimulatory molecule) [47]. Blockade of CTLA-4 has been shown to augment T-cell activation and proliferation, which promotes anti-tumor immune response. This process may also promote autoimmune reactions similar to complications caused by PD-, and PD-L1 antibodies.

There was an increased inflammation, enhanced serum markers of immune damage, and increased infiltration of CD8^+^ T cells in PD-1^−^/CD8^+^ T cells compared to PD-1^+^/CD8^+^ T cells in a CD8^+^ T cell-mediated adoptive transfer model. PD-1 protects against inflammation and myocyte damage in T cell-mediated myocarditis [48].

One possible pathophysiologic mechanism of ICI-related myocarditis is that cardiac myocytes may share targeted antigens with the tumor, therefore becoming targets of activated T-lymphocytes resulting in lymphocytic infiltration of the myocardium [11]. However, this hypothesis seems unlikely due to the role of CTLA-4, PD-1, and PD-L1 molecules in immunology. The blockage of these proteins causes the overactivation of lymphocytes and autoimmune reaction.

Under histological examination, we can observe infiltration of CD8^+^ T-cells, CD68^+^ macrophages, and signs of myocardial fibrosis [29,49]. Additionally, pericardial effusion may develop both early and late during treatment and be symptomatic with tamponade, or occur without any symptoms. In the case of pericardial effusion, heart biopsies show infiltration of T-lymphocytes, mostly CD4^+^ [50].

### 3.3. Clinical Presentation of ICI-Related Myocarditis

The signs and symptoms of ICI-related myocarditis are unspecific. At treatment initiation no specific biomarkers of potential autoimmune risk may be evaluated directly, but initial abnormal troponin levels, NT-proBNP and CK-MB indicating heart muscle damage may be helpful in defining patients at risk of cardiac complications. As in our patient, the myocarditis was manifested by physical deterioration, headache, and shortness of breath initially. Despite the mild presentation of complaints, the course was fulminant, and the patient died less than 24 h after admission. Due to the risk of acute course, it is important to be aware of this rare complication of immunotherapy that can accelerate clinical diagnosis. In our case, the first symptoms occurred six days earlier, but it was ignored by the general practitioner which delayed the beginning of treatment of myocarditis.

At presentation, most patients present with an abnormal electrocardiogram (ECG) and cardiovascular symptoms, usually with elevated troponin, creatinine kinase MB or/and B-type natriuretic peptide (BNP)/N-terminal prohormone of BNP (NT-pro BNP) levels. It has been discussed, whether troponin T remains a good biochemical marker of myocarditis, as it is found in fetal skeletal muscle and it might be less cardiac-specific than troponin I in the presence of neuromuscular pathologies such as ICI-associated myositis [51]. In one study, it was observed that 94% of patients with ICI-related myocarditis (*n* = 35) had an increased troponin level [12]. In our case, a high level of troponin was noted, but the significant increasing tendency was not observed. ECG changes in myocarditis are not specific and include ST-segment and T-wave abnormalities, conduction alternations such as bundle-branch blocks and AV conduction delays, and atrial and ventricular tachyarrhythmias. PR-segment depression and ST-segment elevation without reciprocal changes were observed with pericardial inflammation [52]. In our patient, there were no indications for interventional treatment according to ESC guidelines [53]. Interestingly, the initiation of immunotherapy can induce changes in ECG parameters in melanoma patients. It was recently evaluated by experts from “Essen Cardio-oncology Registry” (ECoR). They showed that heart rate, PR time, QRS, and QTc did not differ when comparing values before and after therapy started. However, QTd was prolonged after therapy started (32 ± 16 ms vs. 47 ± 19 ms, *n* = 41, *p* < 0.0001). Subgroup analyses revealed prolonged QTd in patients that received combination immunotherapy with ipilimumab and nivolumab (31 ± 14 ms vs. 50 ± 14 ms, *n* = 21, *p* < 0.0001), while QTd in patients with anti-programmed death 1 (PD-1) inhibitor monotherapy did not change after therapy started. QTd is prolonged in patients under ICI combination therapy, potentially signaling an increased susceptibility to ventricular arrhythmias [54].

Echocardiographic examinations in the early stages of myocarditis are normal but can have transient wall thickening with edema, impaired left and/or right ventricular functions with preserved EF, or ventricular dilation. Traditionally, myocarditis unrelated to an ICI presenting with a preserved EF is a comparatively benign entity; in contrast, data from various research groups have shown that myocarditis related to an ICI is not [55,56]. As in our case, despite slightly reduced contractility of left ventricle with basal inferior wall hypokinesis, was symptomatic.

Cardiac MRI can show regional or globally increased T2-weighted signal with gadolinium enhancement and/or myocardial injury through T1-based markers (including the presence of late gadolinium enhancement following a non-ischemic distribution) [15,34]. Moreover, cardiac MRI may quantitate tissue injury, including edema, hyperemia, and fibrosis, and can support the diagnosis of myocarditis (Lake Louis criteria) [57,58]. Based on the Lake Louis criteria, when two or more of the three criteria are positive, myocardial inflammation can be predicted with a diagnostic accuracy of 78% in patients with myocarditis unrelated to ICI treatment The sensitivity and specificity of these criteria should be verified in this group of patients but preliminary data suggest that they can be useful in this indication [12]. Also, FDG-PET-CT imaging might be beneficial in confirming the diagnosis [59]. However, according to ESC guidelines EMB is still the gold standard, and should be performed in any case of suspicion, especially among patients with a life-threatening arrhythmia, LV dysfunction that does not improve 4–5 days after onset of symptoms, exacerbating LV dysfunction within 4–5 days after onset of symptoms, and recurrent myocarditis [39,60]. In our case, due to the inaccessibility to EMB, it was not performed before the beginning of the immunosuppressive treatment. It seems that the cardiac MRI or PET-CT may be used among patients who undergo EMB but it should be verified in clinical studies.

### 3.4. Other Specific Organ Toxicities Associated with ICI

In a series by Heinzerling et al., five of the eight patients’ (63%) other organ systems were also affected by immune-related side effects, including autoimmune thyroiditis, uveitis, colitis, hepatitis, and hypophysitis [21]. Similar observations were made by Ansari-Gilani et al. [61]. Hepatitis was also associated with myocarditis in a case reported by Samara et al. [62]. Patients who develop myasthenia gravis as the irAE, have been reported to have a 16–37% chance of also having myocardial changes [63,64]. In Japanese patients treated with immune checkpoint inhibitors, among twelve patients who developed myasthenia gravis, three presented with myocarditis [65]. Also, Bawek et al. presented a case with myocarditis concomitant with myasthenia gravis; in addition, the patient was also diagnosed with myositis and hepatitis [66]. Moslehi et al. reported myositis/rhabdomyolysis, myasthenia gravis, colitis, and severe cutaneous events (Stevens–Johnson syndrome, bullous pemphigus, and skin necrosis) as the most frequent concurrent irAE [14]. A recent systemic review showed that myocarditis and myositis have a propensity to occur together, and ICI-induced myositis can be associated with myasthenia gravis in up to 40% of patients [64]. Another concomitant neurologic irAE is axonal polyradiculoneuropathy, as reported by Diamantopoulos et al. [67]. Koelzer et al., reported a case of lymphocytic myocarditis as a part of systemic inflammation associated with ipilimumab and nivolumab administration [49]. Some authors suggest that myocarditis is associated with response to therapy [68]. However, a case of myocarditis with concomitant hyper-progression was also described [69]. It is worth taking into account that not all cardiac and non-cardiac manifestations occurring under ICI therapy are drug-related adverse events, therefore differential diagnoses must be considered in order to avoid unnecessary cessation of ICI treatment, which may have a major impact on overall patient prognosis [70].

### 3.5. Risk Factors for ICI-Related Myocarditis

The combination of ICI therapy is the most established risk factor. It was shown that the risk of myocarditis increased by 4.74 among patients who received combination (nivolumab + ipilimumab) therapy in comparison to monotherapy (nivolumab) [11]. In a case series by Heinzerling et al., pre-existing pathology, including cardiac pathology or peripheral arterial disease, was present in the majority of the patients (five out of eight), but patients were free of symptoms when starting checkpoint inhibitor therapy [21]. Mahmood et al., in a retrospective study, implicated diabetes and perhaps pre-existing heart disease as a risk factor for myocarditis [12]. Johnson et al. (2016) described two fatal cases with fulminant myocarditis associated with the combination of ipilimumab with nivolumab, and both patients were hypertensive but did not have other cardiac risk factors [11]. Other authors also report pre-existing hypertension [18,71,72]. Some reported patients also presented with coronary bypass implanted after MI, past cerebrovascular accidents, or aortic aneurysms [18]. In a single-center study by Drobni et al., patients on ICIs were at a threefold increased risk of MI and stroke due to increased aortic atherosclerotic plaque burden, as compared with non-ICI patients [73]. Some authors suggest that patients with baseline organ dysfunction, commonly excluded from clinical trials, represent a population that may be more susceptible to adverse events [74]. However, in the population presented by Shah et al. (2020), performance status at the time of initiation of immunotherapy treatment did not show a significant association with toxicity [75]. Johnson et al. presented a series of 30 patients with a range of pre-existing autoimmune disorders, treated with ipilimumab for advanced melanoma. Interestingly, ipilimumab was active (20% response rate), despite the fact that 43% of patients were on immunosuppressants at the time of ipilimumab commencement. While 27% of patients experienced a flare of their autoimmune disorder and 33% experienced grade 3–5 irAEs, no cardiac toxicity was observed [76].

### 3.6. Treatment of ICI-Related Myocarditis

**Figure 4:**
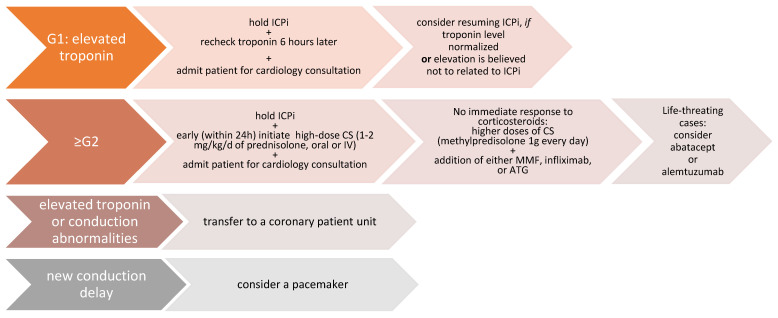
Management of cardiovascular toxicities, including myocarditis, pericarditis, arrhythmias, impaired ventricular function with heart failure, and vasculitis. Based on [78]; G1—grade 1, G2—grade 2 (see Table 3), ICIs—immune checkpoints inhibitor, CS—corticosteroid, MMF—mycophenolate mofetil, ATG—anti-thymocyte globulin.

The summary of recommended treatment guidelines is presented in figure (Figure 4).

The consensus for the initial steroid dose is an equivalent dose of (methyl)prednisolone 1–2 mg/kg for grade 2 or higher cardiotoxicity (Table 2) [77,78]. CTLA-4 agonist abatacept, alemtuzumab, tocilizumab, rituximab, anti-thymocyte globulin, intravenous immunoglobulins, tacrolimus, mycophenolate, azathioprine, methotrexate, cyclophosphamide, and infliximab or plasmapheresis have been used in steroid-refractory cases of myocarditis, although infliximab is not preferred at higher dosing given its association with cardiac failure [34,64,79]. Matzen et al. compared fatality in identified cases and 55% treated with high-dose steroids only were fatal versus 43% fatality in cases treated with other immunosuppressive agents [16]. There is currently no consensus on the optimal immunosuppression treatment sequence, combination, and duration in steroid-refractory cases. Moreover, it should be taken into account that lymphocyte depletion associated with immunosuppressive therapy may induce the unleashing of aggressive melanoma and the progression of the disease [80]. A recent study showed that the blockade of TNFα may lead to preventing the manifestation of ICI-related cardiotoxicity [81]. Balanescu et al. (2020) described for the first time the successful rechallenge with ICI after cured ICI-induced myocarditis. The patient, who developed myocarditis after the combination of nivolumab and ipilimumab, was able to reinitiate monotherapy with nivolumab at short notice [82].

**Table 3:** Grading of cardiovascular toxicities, including myocarditis, pericarditis, arrhythmias, impaired ventricular function with heart failure, and vasculitis. Based on [78].

G1	Abnormal cardiac biomarker testing without symptoms and with no ECG abnormalities
G2	Abnormal cardiac biomarker testing with mild symptoms or new ECG abnormalities without conduction delay
G3	Abnormal cardiac biomarker testing with either moderate symptoms or new conduction delay
G4	Moderate to severe decompensation, IV medication or intervention required, life-threatening conditions

### 3.7. Follow-Up and Surveillance of Patients

The cardiac history should be taken from all patients before initiation of ICI therapy. Additionally, the measurement of troponin level and ECG should be performed [47]. The patients with concomitant cardiac diseases or initial troponinemia have non-ICI-related myocardial damage and are more susceptible to myocarditis. In one study, the authors measured the troponin level one time per week for 6 weeks (from the first dose of ICI) [13]. In any case of elevation of this cardiac biomarker during follow-up, the patient should be consulted with a cardiologist and an echocardiogram should be done. A cardiac biopsy should be considered if there is any sign of myocarditis in an echocardiogram or cardiac MRI [13]. However, in many cases elevation of troponin during immunotherapy can be not associated with myocarditis. Melanoma was reported to be the second most frequent cancer associated with cardiac metastases, after mesothelioma, so the progression of the disease and heart failure related to increasing after-load should be always taken into consideration [83,84]. Also, Kurzhals et al. (2021) reported elevations of serum troponin T levels in 28% of patients with advanced skin cancer prior to the beginning of the therapy, and the pre-therapeutic elevated troponin T concentrations were not associated with the development of myocarditis [85]. However, if and how the extent to which metastatic melanoma *per se* is associated with increased serum troponin T concentrations needs to be confirmed in larger studies. The potential risk of long-term cardiotoxicity has not been established yet, due to the lack of sufficient long-term data as most of these agents have been approved in the last few years [40].

## 4. Conclusions

Cardiotoxic AEs, initially reported as very rare, are more often becoming recognized as the use of immune checkpoint inhibitors is expanded outside clinical trials in general everyday practice. At the start of immunotherapy treatment, no specific biomarkers of potential autoimmune cardiac risk are known at this point in time, but initial abnormal troponin, NT-proBNP and CK/CK-MB levels indicating heart muscle damage may be helpful in defining patients at risk. As in our patient, the myocarditis was manifested by physical deterioration, ECG abnormalities or chest pain.

Monitoring of patients with pre-existing cardiac disorders and risk factors is essential in everyday practice and may accelerate the diagnosis and rapid initiation of treatment for myocarditis. Special attention needs to be focused on patients who have already experienced one immune-related adverse event such as colitis, pneumonitis, hepatitis, neurotoxicity, and hyper- or hypothyroidism. In any case of cardiac signs or symptoms, prompt evaluation is essential which can ensure fast initiation of treatment for myocarditis. It can reduce the risk of death due to this complication and allow reinitiating the immunotherapy after curing the myocarditis in some cases. More research on autoimmunity biomarkers is needed. Prospective translational trials could help to define patients at risk. Serum-based biomarkers that are easily obtained in routine practice should be considered.

## Figures and Tables

**Table 1:** Changes in selected laboratory parameters during immunotherapy. ALT-alanine transaminase, ASPAT-aspartate transaminase, CK-creatine kinase, CRP-C-reactive protein, LDH-lactate dehydrogenase.

Parameter	Reference Range	Before Initiation of Immunotherapy	After 3rd Course of Nivolumab	On Admission to the 4th Course
ASPAT	<50 (IU/L)	16	71	178
ALT	<50 (IU/L)	10	49	207
CK	<171 (IU/L)	60	-	2194
CRP	<5 (mg/L)	2.8	-	6.1
LDH	<247 (IU/L)	222	377	943

**Table 2:** Criterias of diagnosis of myocarditis. Based on [35].

Definitive diagnosis	Histology—EMB (according to Dallas criteria [38])	Active myocarditis: an inflammatory infiltrate of the myocardium with necrosis and/or degeneration of adjacent myocytes not typical of the ischemic damage associated with coronary artery disease. Borderline myocarditis: sparse inflammatory infiltrate or myocytes without evident injury.
Diagnosis of clinically suspected myocarditis [35]: ≥1 of the clinical presentations of myocarditis and ≥1 diagnostic criteria (if the patient is asymptomatic, ≥2 diagnostic criteria are required)	Clinical presentations	- acute chest pain (pericarditis or pseudo-ischemic) - new-onset (days up to three months) or worsening of dyspnea at rest or exercise, and/or fatigue, with or without left and/or right HF signs - palpitation, and/or unexplained arrhythmia symptoms and/or syncope, and/or aborted sudden cardiac death - unexplained cardiogenic shock
	Diagnostic criteria	- ECG/Holter stress test features –first to third degree AV block or bundle branch block, ST/T wave change (ST elevation or T wave inversion), sinus arrest, VT or VF, asystole, AF, significantly reduced R wave height, IVCD (widened QRS complex), abnormal Q waves, low voltage, frequent premature beats, or SVT - elevated troponin T or troponin I. - functional and structural abnormalities on cardiac imaging (echocardiogram, angiogram, or CMR—new, otherwise unexplained abnormality of LV and/or RV function (regional wall motion abnormality or global systolic or diastolic dysfunction) - tissue characterization by CMR—the presence of updated Lake Louse criteria suggests myocarditis

EMB—endomyocardial biopsy, HF—heart failure, AV—atrioventricular block, VT—ventricular tachycardia, VF—ventricular fibrillation, AF—atrial fibrillation, IVCD—intraventricular conduction delay, SVT—supraventricular tachycardia.

## Data Availability

All data are available for research cooperation purposes from the PI of the study upon DTA approval.

## Supplementary Materials

The following supporting information can be downloaded at: https://www.mdpi.com/article/10.3390/jcm11175182/s1, Table S1. Description of case of cardiac complications of immunotherapy.

## References

[1] Kennedy L.B., Salama A. (2020). A review of cancer immunotherapy toxicity. CA A Cancer J. Clin..

[2] Jia X.-H., Geng L.-Y., Jiang P.-P., Xu H., Nan K.-J., Yao Y., Jiang L.-L., Sun H., Qin T.-J., Guo H. (2020). The biomarkers related to immune related adverse events caused by immune checkpoint inhibitors. J. Exp. Clin. Cancer Res..

[3] Weidhaas J., Marco N., Scheffler A.W., Kalbasi A., Wilenius K., Rietdorf E., Gill J., Heilig M., Desler C., Telesca D. (2022). Germline biomarkers predict toxicity to anti-PD1/PDL1 checkpoint therapy. J. Immunother. Cancer.

[4] Wang D.Y., Salem J.E., Cohen J.V., Chandra S., Menzer C., Ye F., Zhao S., Das S., Beckermann K.E., Johnson D.B. (2018). Fatal Toxic Effects Associated With Immune Checkpoint Inhibitors: A Systematic Review and Meta-analysis. JAMA Oncol..

[5] Zimmer L., Goldinger S.M., Hofmann L., Loquai C., Ugurel S., Thomas I., Schmidgen M., Gutzmer R., Utikal J., Heinzerling L. (2016). Neurological, respiratory, musculoskeletal, cardiac and ocular side-effects of anti-PD-1 therapy. Eur. J. Cancer.

[6] Oldfield K., Jayasinghe R., Niranjan S., Chadha S. (2021). Immune checkpoint inhibitor-induced takotsubo syndrome and diabetic ketoacidosis: Rare reactions. BMJ Case Rep..

[7] Voskens C.J., Goldinger S.M., Loquai C., Robert C., Kaehler K.C., Al E., Dummer R. (2013). The price of tumor control: An analysis of rare side effects of anti-CTLA-4 therapy in metastatic melanoma from the ipilimumab network. PLoS One.

[8] Tomita Y., Sueta D., Kakiuchi Y., Saeki S., Saruwatari K., Sakata S., Jodai T., Migiyama Y., Akaike K., Hirosako S. (2017). Acute coronary syndrome as a possible immune-related adverse event in a lung cancer patient achieving a complete response to anti-PD-1 immune checkpoint antibody. Ann. Oncol..

[9] Heery C.R., Coyne G.H.O., Madan R.A., Schlom J., Von Heydebreck A., Cuillerot J.-M., Sabzevari H., Gulley J.L. (2014). Phase I open-label, multiple ascending dose trial of MSB0010718C, an anti-PD-L1 monoclonal antibody, in advanced solid malignancies. J. Clin. Oncol..

[10] Tadokoro T., Keshino E., Makiyama A., Sasaguri T., Ohshima K., Katano H., Mohri M. (2016). Acute Lymphocytic Myocarditis with Anti-PD-1 Antibody Nivolumab. Circ. Heart Fail..

[11] Johnson D.B., Balko J.M., Compton M.L., Chalkias S., Gorham J., Xu Y., Hicks M., Puzanov I., Alexander M., Moslehi J.J. (2016). Fulminant Myocarditis with Combination Immune Checkpoint Blockade. N. Engl. J. Med..

[12] Mahmood S.S., Fradley M.G., Cohen J.V., Nohria A., Reynolds K.L., Heinzerling L.M., Sullivan R., Damrongwatanasuk R., Chen C., Neilan T.G. (2018). Myocarditis in Patients Treated With Immune Checkpoint Inhibitors. J. Am. Coll. Cardiol..

[13] Salem J.E., Manouchehri A., Moey M., Lebrun-Vignes B., Bastarache L., Pariente A., Gobert A., Spano P., Balko J., Moslehi J.J. (2018). Cardiovascular toxicities associated with immune checkpoint inhibitors: An observational, retrospective, pharmacovigilance study. Lancet Oncol..

[14] Moslehi J.J., Salem J.E., Sosman J.A., Lebrun-Vignes B., Johnson D.B. (2018). Increased reporting of fatal immune checkpoint inhibitor-associated myocarditis. Lancet.

[15] Tajiri K., Aonuma K., Sekine I. (2017). Immune checkpoint inhibitor-related myocarditis. Jpn. J. Clin. Oncol..

[16] Matzen E., Bartels L.E., Løgstrup B., Horskær S., Stilling C., Donskov F. (2021). Immune checkpoint inhibitor-induced myocarditis in cancer patients: A case report and review of reported cases. Cardio-Oncol..

[17] D’Souza M., Nielsen D., Svane I.M., Iversen K., Rasmussen P.V., Madelaire C., Fosbøl E., Køber L., Gustafsson F., Andersson C. (2020). The risk of cardiac events in patients receiving immune checkpoint inhibitors: A nationwide Danish study. Eur. Heart J..

[18] Scard C., Nguyen J.-M., Varey E., Moustaghfir I., Khammari A., Dreno B. (2021). Cardiac adverse events associated with anti-PD-1 therapy in patients treated for advanced melanoma: Relevance of dosing troponin T levels. Eur. J. Dermatol..

[19] Qin Q., Patel V.G., Wang B., Mellgard G., Gogerly-Moragoda M., Zhong X., Parikh A.B., Leiter A., Gallagher E.J., Galsky M.D. (2020). Type, timing, and patient characteristics associated with immune-related adverse event development in patients with advanced solid tumors treated with immune checkpoint inhibitors. J. Clin. Oncol..

[20] Jain P., Bugarin J.G., Guha A., Jain C., Patil N., Shen T., Stanevich I., Nikore V., Margolin K., Dowlati A. (2021). Cardiovascular adverse events are associated with usage of immune checkpoint inhibitors in real-world clinical data across the United States. ESMO Open.

[21] Heinzerling L., Ott P.A., Hodi F.S., Husain A.N., Tajmir-Riahi A., Tawbi H., Pauschinger M., Gajewski T.F., Lipson E.J., Luke J.J. (2016). Cardiotoxicity associated with CTLA4 and PD1 blocking immunotherapy. J. Immunother. Cancer.

[22] Rahouma M., Karim N.A., Baudo M., Yahia M., Kamel M., Eldessouki I., Abouarab A., Saad I., Elmously A., Gaudino M. (2019). Cardiotoxicity with immune system targeting drugs: A meta-analysis of anti-PD/PD-L1 immunotherapy randomized clinical trials. Immunotherapy.

[23] Eggermont A.M., Chiarion-Sileni V., Grob J.J., Dummer R., Wolchok J.D., Schmidt H., Hamid O., Robert P., Richards J., Testori A. (2015). Adjuvant ipilimumab versus placebo after complete resection of high-risk stage III melanoma (EORTC 18071): A randomised, double-blind, phase 3 trial. Lancet Oncol..

[24] Saibil S.D., Bonilla L., Majeed H., Sotov V., Hogg D., Chappell M.A., Cybulsky M., Butler M.O. (2019). Fatal Myocarditis and Rhabdomyositis in a Patient with Stage Iv Melanoma Treated with Combined Ipilimumab and Nivolumab. Curr. Oncol..

[25] Yamaguchi S., Morimoto R., Okumura T., Yamashita Y., Haga T., Kuwayama T., Yokoi T., Hiraiwa H., Kondo T., Sugiura Y. (2018). Late-Onset Fulminant Myocarditis with Immune Checkpoint Inhibitor Nivolumab. Can. J. Cardiol..

[26] Arangalage D., Delyon J., Lermuzeaux M., Ekpe K., Ederhy S., Pages C., Lebbé C. (2017). Survival After Fulminant Myocarditis Induced by Immune-Checkpoint Inhibitors. Ann. Intern. Med..

[27] Geisler B.P., Raad R.A., Esaian D., Sharon E., Schwartz D.R. (2015). Apical ballooning and cardiomyopathy in a melanoma patient treated with ipilimumab: A case of takotsubo-like syndrome. J. Immunother. Cancer.

[28] Yun S., Vincelette N.D., Mansour I., Hariri D., Motamed S. (2015). Late Onset Ipilimumab-Induced Pericarditis and Pericardial Effusion: A Rare but Life Threatening Complication. Case Rep. Oncol. Med..

[29] Läubli H., Balmelli C., Bossard M., Pfister O., Glatz K., Zippelius A. (2015). Acute heart failure due to autoimmune myocarditis under pembrolizumab treatment for metastatic melanoma. J. Immunother. Cancer.

[30] Naidoo J., Wang X., Woo K.M., Iyriboz T., Halpenny D., Cunningham J., Chaft J., Segal N., Callahan M., Hellmann M.D. (2017). Pneumonitis in Patients Treated With Anti-Programmed Death-1/Programmed Death Ligand 1 Therapy. J. Clin. Oncol..

[31] Hofmann L., Forschner A., Loquai C., Goldinger S.M., Zimmer L., Ugurel S., Schmidgen M.I., Gutzmer R., Utikal J.S., Göppner D. (2016). Cutaneous, gastrointestinal, hepatic, endocrine, and renal side-effects of anti-PD-1 therapy. Eur. J. Cancer.

[32] Dearden H., Au L., Wang D.Y., Zimmer L., Eroglu Z., Smith J.L., Cuvietto M., Khoo C., Atkinson V., Lo S. (2021). Hyperacute toxicity with combination ipilimumab and anti-PD1 immunotherapy. Eur. J. Cancer.

[33] Shulgin B., Kosinsky Y., Omelchenko A., Chu L., Mugundu G., Aksenov S., Pimentel R., DeYulia G., Kim G., Peskov K. (2020). Dose dependence of treatment-related adverse events for immune checkpoint inhibitor therapies: A model-based meta-analysis. OncoImmunology.

[34] Arangalage D., Degrauwe N., Michielin O., Monney P., Özdemir B.C. (2021). Pathophysiology, diagnosis and management of cardiac toxicity induced by immune checkpoint inhibitors and BRAF and MEK inhibitors. Cancer Treat. Rev..

[35] Caforio A.L., Pankuweit S., Arbustini E., Basso C., Gimeno-Blanes J., Felix S.B., Fu M., Heliö T., Heymans S., Elliott P.M. (2013). Current state of knowledge on aetiology, diagnosis, management, and therapy of myocarditis: A position statement of the European Society of Cardiology Working Group on Myocardial and Pericardial Diseases. Eur. Heart J..

[36] Cooper L.T. (2009). Myocarditis. New Engl. J. Med..

[37] Richardson P. (1996). Report of the 1995 World Health Organization/International Society and Federation of Cardiology Task Force on the Definition and Classification of cardiomyopathies. Circulation.

[38] Aretz H.T., Billingham M.E., Edwards W.D., Factor S.M., Fallon J., Fenoglio J.J., Olsen E.G., Schoen F.J. (1987). Myocarditis. A histopathologic definition and classification. Am. J. Cardiovasc. Pathol..

[39] Cooper L.T., Baughman K.L., Feldman A.M., Frustaci A., Jessup M., Kuhl U., Levine G., Narula J., Starling R.C., Virmani R. (2007). The role of endomyocardial biopsy in the management of cardiovascular disease: A scientific statement from the American Heart Association, the American College of Cardiology, and the European Society of Cardiology Endorsed by the Heart Failure Society of America and the Heart Failure Association of the European Society of Cardiology. Eur. Heart J..

[40] Lobenwein D., Kocher F., Dobner S., Gollmann-Tepeköylü C., Holfeld J. (2020). Cardiotoxic mechanisms of cancer immunotherapy–A systematic review. Int. J. Cardiol..

[41] Grabie N., Gotsman I., Dacosta R., Pang H., Stavrakis G., Butte M.J., Keir M.E., Freeman G.J., Sharpe A.H., Lichtman A.H. (2007). Endothelial Programmed Death-1 Ligand 1 (PD-L1) Regulates CD8 + T-Cell–Mediated Injury in the Heart. Circulation.

[42] Seko Y., Yagita H., Okumura K., Azuma M., Nagai R. (2007). Roles of programmed death-1 (PD-1)/PD-1 ligands pathway in the development of murine acute myocarditis caused by coxsackievirus B3. Cardiovasc. Res..

[43] Li S., Tajiri K., Murakoshi N., Xu D., Yonebayashi S., Okabe Y., Yuan Z., Feng D., Inoue K., Aonuma K. (2021). Programmed Death-Ligand 2 Deficiency Exacerbates Experimental Autoimmune Myocarditis in Mice. Int. J. Mol. Sci..

[44] Nishimura H., Okazaki T., Tanaka Y., Nakatani K., Hara M., Matsumori A., Sasayama S., Mizoguchi A., Hiai H., Minato N. (2001). Autoimmune Dilated Cardiomyopathy in PD-1 Receptor-Deficient Mice. Science.

[45] Nishimura H., Nose M., Hiai H., Minato N., Honjo T. (1999). Development of Lupus-like Autoimmune Diseases by Disruption of the PD-1 Gene Encoding an ITIM Motif-Carrying Immunoreceptor. Immunity.

[46] Tay W.T., Fang Y.-H., Beh S.T., Liu Y.-W., Hsu L.-W., Yen C.-J., Liu P.-Y. (2020). Programmed Cell Death-1: Programmed Cell Death-Ligand 1 Interaction Protects Human Cardiomyocytes Against T-Cell Mediated Inflammation and Apoptosis Response In Vitro. Int. J. Mol. Sci..

[47] Hu J.R., Florido R., Lipson E.J., Naidoo J., Ardehali R., Tocchetti C.G., Lyon A.R., Padera R., Johnson D., Moslehi J. (2019). Cardiovascular toxicities associated with immune checkpoint inhibitors. Cardiovasc. Res..

[48] Tarrio M.L., Grabie N., Bu D.-X., Sharpe A.H., Lichtman A.H. (2012). PD-1 Protects against Inflammation and Myocyte Damage in T Cell-Mediated Myocarditis. J. Immunol..

[49] Koelzer V.H., Rothschild S.I., Zihler D., Wicki A., Willi B., Willi N., Voegeli M., Cathomas G., Zippelius A., Mertz K.D. (2016). Systemic inflammation in a melanoma patient treated with immune checkpoint inhibitors—an autopsy study. J. Immunother. Cancer.

[50] Saade A., Mansuet-Lupo A., Arrondeau J., Thibault C., Mirabel M., Goldwasser F., Oudard S., Weiss L. (2019). Pericardial effusion under nivolumab: Case-reports and review of the literature. J. Immunother Cancer.

[51] Ang E., Mweempwa A., Heron C., Ahn Y., Rivalland G., Ha L.Y., Deva S. (2021). Cardiac Troponin I and T in Checkpoint Inhibitor–associated Myositis and Myocarditis. J. Immunother..

[52] Arponen O., Skyttä T. (2020). Immune checkpoint inhibitor-induced myocarditis not visible with cardiac magnetic resonance imaging but detected with PET-CT: A case report. Acta Oncol..

[53] Ibanez B., James S., Agewall S., Antunes M.J., Bucciarelli-Ducci C., Bueno H., Caforio A., Crea F., Goudevenos F., Widimský P. (2018). 2017 ESC Guidelines for the management of acute myocardial infarction in patients presenting with ST-segment elevation: The Task Force for the management of acute myocardial infarction in patients presenting with ST-segment elevation of the European Society of Cardiology (ESC). Eur. Heart J..

[54] Pohl J., Mincu R.-I., Mrotzek S.M., Hinrichs L., Michel L., Livingstone E., Zimmer L., Wakili R., Schadendorf D., Rassaf T. (2020). ECG Changes in Melanoma Patients Undergoing Cancer Therapy–Data from the ECoR Registry. J. Clin. Med..

[55] Ammirati E., Cipriani M., Moro C., Raineri C., Pini D., Sormani P., Mantovani R., Varrenti M., Pedrotti P., delle Miocarditi R.L. (2018). Clinical Presentation and Outcome in a Contemporary Cohort of Patients With Acute Myocarditis: Multicenter Lombardy Registry. Circulation.

[56] Awadalla M., Mahmood S.S., Groarke J.D., Hassan M.Z., Nohria A., Rokicki A., Murphy S., Mercaldo N., Zhang L., Neilan T.G. (2020). Global Longitudinal Strain and Cardiac Events in Patients With Immune Checkpoint Inhibitor-Related Myocarditis. J. Am. Coll. Cardiol..

[57] Friedrich M.G., Sechtem U., Schulz-Menger J., Holmvang G., Alakija P., Cooper L.T., White J.A., Abdel-Aty H., Gutberlet M., Prasad S. (2009). Cardiovascular Magnetic Resonance in Myocarditis: A JACC White Paper. J. Am. Coll. Cardiol..

[58] Olimulder M.A., van Es J., Galjee M.A. (2009). The importance of cardiac MRI as a diagnostic tool in viral myocarditis-induced cardiomyopathy. Neth. Heart J..

[59] Nensa F., Kloth J., Tezgah E., Poeppel T.D., Heusch P., Goebel J., Nassenstein K., Schlosser T. (2018). Feasibility of FDG-PET in myocarditis: Comparison to CMR using integrated PET/MRI. J. Nucl. Cardiol..

[60] Hazebroek M.R., Everaerts K., Heymans S. (2014). Diagnostic approach of myocarditis: Strike the golden mean. Neth. Heart J..

[61] Ansari-Gilani K., Tirumani S.H., Smith D.A., Nelson A., Alahmadi A., Hoimes C.J., Ramaiya N.H. (2020). Myocarditis associated with immune checkpoint inhibitor therapy: A case report of three patients. Emerg. Radiol..

[62] Samara Y., Yu C.L., Dasanu C.A. (2018). Acute autoimmune myocarditis and hepatitis due to ipilimumab monotherapy for malignant melanoma. J. Oncol. Pharm. Pract..

[63] Fukasawa Y., Sasaki K., Natsume M., Nakashima M., Ota S., Watanabe K., Takahashi Y., Kondo F., Kozuma K., Seki N. (2017). Nivolumab-Induced Myocarditis Concomitant with Myasthenia Gravis. Case Rep. Oncol..

[64] Pathak R., Katel A., Massarelli E., Villaflor V.M., Sun V., Salgia R. (2021). Immune Checkpoint Inhibitor–Induced Myocarditis with Myositis/Myasthenia Gravis Overlap Syndrome: A Systematic Review of Cases. Oncologist.

[65] Suzuki S., Ishikawa N., Konoeda F., Seki N., Fukushima S., Takahashi K., Uhara H., Hasegawa Y., Inomata S., Matsui M. (2017). Nivolumab-related myasthenia gravis with myositis and myocarditis in Japan. Neurology.

[66] Bawek S.J., Ton R., McGovern-Poore M., Khoncarly B., Narvel R. (2021). Nivolumab-Induced Myasthenia Gravis Concomitant with Myocarditis, Myositis, and Hepatitis. Cureus.

[67] Diamantopoulos P.T., Tsatsou K., Benopoulou O., Bonou M., Anastasopoulou A., Mastrogianni E., Gogas H. (2020). Concomitant development of neurologic and cardiac immune-related adverse effects in patients treated with immune checkpoint inhibitors for melanoma. Melanoma Res..

[68] Shalata W., Peled N., Gabizon I., Saleh O.A., Kian W., & Yakobson A. (2020). Associated Myocarditis: A Predictive Factor for Response?. Case Rep. Oncol..

[69] Barham W., Guo R., Park S.S., Herrmann J., Dong H., Yan Y. (2021). Case Report: Simultaneous Hyperprogression and Fulminant Myocarditis in a Patient with Advanced Melanoma Following Treatment With Immune Checkpoint Inhibitor Therapy. Front. Immunol..

[70] Arangalage D., Pavon A.G., Özdemir B.C., Michielin O., Schwitter J., Monney P. (2021). Acute cardiac manifestations under immune checkpoint inhibitors—beware of the obvious: A case report. Eur. Heart J.-Case Rep..

[71] Fazel M., Jedlowski P.M. (2019). Severe Myositis, Myocarditis, and Myasthenia Gravis with Elevated Anti-Striated Muscle Antibody following Single Dose of Ipilimumab-Nivolumab Therapy in a Patient with Metastatic Melanoma. Case Rep. Immunol..

[72] Wakefield C., Shultz C., Patel B., Malla M. (2021). Life-threatening immune checkpoint inhibitor-induced myocarditis and myasthenia gravis overlap syndrome treated with abatacept: A case report. BMJ Case Rep..

[73] Drobni Z.D., Alvi R.M., Taron J., Zafar A., Murphy S.P., Rambarat P.K., Mosarla R.C., Lee C., Zlotoff D.A., Raghu V.K. (2020). Association Between Immune Checkpoint Inhibitors with Cardiovascular Events and Atherosclerotic Plaque. Circulation.

[74] Kanz B.A., Pollack M.H., Johnpulle R., Puzanov I., Horn L., Morgans A., Sosman J.A., Rapisuwon S., Conry R.M., Eroglu Z. (2016). Safety and efficacy of anti-PD-1 in patients with baseline cardiac, renal, or hepatic dysfunction. J. Immunother. Cancer.

[75] Shah K.P., Song H., Ye F., Moslehi J.J., Balko J.M., Salem J.-E., Johnson D.B. (2020). Demographic Factors Associated with Toxicity in Patients Treated with Anti–Programmed Cell Death-1 Therapy. Cancer Immunol. Res..

[76] Johnson D.B., Sullivan R.J., Ott P.A., Carlino M.S., Khushalani N.I., Ye F., Buchbinder E., Mudigonda T., Spencer K., Clark J.I. (2016). Ipilimumab Therapy in Patients With Advanced Melanoma and Preexisting Autoimmune Disorders. JAMA Oncol..

[77] Haanen J.B.A.G., Carbonnel F., Robert C., Kerr K.M., Peters S., Larkin J., Jordan K. (2017). Management of toxicities from immunotherapy: ESMO Clinical Practice Guidelines for diagnosis, treatment and follow-up. Ann. Oncol..

[78] Schneider B.J., Naidoo J., Santomasso B.D., Lacchetti C., Adkins S., Anadkat M., Atkins M., Mammen J., Naing A., Bollin K. (2021). Management of Immune-Related Adverse Events in Patients Treated With Immune Checkpoint Inhibitor Therapy: ASCO Guideline Update. J. Clin. Oncol..

[79] Guo C.W., Alexander M., Dib Y., Lau P.K., Weppler A.M., Au-Yeung G., Lee B., Khoo C., Mooney D., Joshi S.B. (2019). A closer look at immune-mediated myocarditis in the era of combined checkpoint blockade and targeted therapies. Eur. J. Cancer.

[80] Norwood T.G., Lenneman C.A., Westbrook B.C., Litovsky S.H., McKee S.B., Conry R.M. (2020). Evolution of Immune Checkpoint Blockade–Induced Myocarditis Over 2 Years. JACC Case Rep..

[81] Michel L., Helfrich I., Hendgen-Cotta U.B., Mincu R.-I., Korste S., Mrotzek S.M., Spomer A., Odersky A., Rischpler C., Herrmann K. (2021). Targeting early stages of cardiotoxicity from anti-PD1 immune checkpoint inhibitor therapy. Eur. Heart J..

[82] Balanescu D.V., Donisan T., Palaskas N., Lopez-Mattei J., Kim P.Y., Buja L.M., McNamara D.M., Kobashigawa J.A., Durand J.-B., Iliescu C.A. (2020). Immunomodulatory treatment of immune checkpoint inhibitor-induced myocarditis: Pathway toward precision-based therapy. Cardiovasc. Pathol..

[83] Benassaia E., Vallet A., Rouleau E., Ederhy S., Robert C. (2021). Troponin increase during immunotherapy: Not always myocarditis. Eur. J. Cancer.

[84] Bussani R., De-Giorgio F., Abbate A., Silvestri F. (2007). Cardiac metastases. J. Clin. Pathol..

[85] Kurzhals J.K., Graf T., Boch K., Grzyska U., Frydrychowicz A., Zillikens D., Terheyden P., Langan E.A. (2021). Serum Troponin T Concentrations Are Frequently Elevated in Advanced Skin Cancer Patients Prior to Immune Checkpoint Inhibitor Therapy: Experience from a Single Tertiary Referral Center. Front. Med..

