# Supplementary material for: Myocarditis Induced by Immunotherapy in Metastatic Melanoma—Review of Literature and Current Guidelines
Source: J Clin Med. 2022 Sep 1;11(17):5182. doi: 10.3390/jcm11175182 (PMC9457343; doi:10.3390/jcm11175182)
Supplement: Supplementary file 1 [file jcm-11-05182-s001.zip › jcm-1810331-supplementary.pdf]

Table S1. Description of cases of cardiac complications of immunotherapy.

Authors, Year	Number of patients, their mean age and gender and type of cancer	Drug and dosage	Signs and symptoms	Outcome
Heinzerling et al. (2016) [1]	8M (67.1), 8 melzanoma	6 ipilimumab (3mg/kg) (1 with nivolumab 1mg/kg; followed by 3mg/kg) , 1 ipilimumab (10mg/kg), 1 pembrolizumab (2mg/kg)	edema, ascites, pleural effusion, dyspnea, decrease of EF	3 deaths, 5 resolved (2 permament decrease of EF)
Johnson et al. (2016) [2]	1M (63), 1 W (65), melanoma	Nivolumab (1mg) and ipilimumab (3mg/kg)	atypical chest pain, dyspnea, and fatigue, profound ST-segment depression, a new intraventricular conduction delay	2 deaths
Saibil et al. (2019) [3]	1M (67), melanoma	Nivolumab (1mg) and ipilimumab (3mg/kg)	fatigue, weakness, and dyspnea, bradycardia and 3rd-degree heart block	death
Yamaguchi et al. (2018) [4]	1M (60), melanoma	Nivolumab (2mg/kg) and ipilimumab (ND)	fatigue and fever without chest pain or dyspnea, ST-segment elevation, decrease of EF (from 70% to 15%)	resolved
Geisler et al. (2015) [5]	1W (83), melanoma	Ipilimumab (3mg/kg)	pruritus, lethargy, and malaise, diarrhoea, progressive dyspnoea, chest pain, akinetic apex, hyperkinetic base and septum (takotsubo-like syndrome), EF=50%,	resolved
Yun et al. [6]	1M (59), melanoma	Ipilimumab (3mg/kg)	acute onset chest pain and dyspnoea, 5cm of jugular venous distension, low QRS complex voltage, ericardial thickening and moderate sized pericardial effusion	resolved
Läubli and Balmelli et al. (2015) [7]	1W (73), melanoma	Pembrolizumab (2mg/kg)	Dyspnoea, congested jugular veins, bilateral rales and lower leg edema, ventricular bigamy, EF=30%	resolved

1. Heinzerling, L., et al., *Cardiotoxicity associated with CTLA4 and PD1 blocking immunotherapy.* 2016. **4**(1): p. 50.
2. Johnson, D.B., et al., *Fulminant Myocarditis with Combination Immune Checkpoint Blockade.* N Engl J Med, 2016. **375**(18): p. 1749-1755.
3. Saibil, S.D., et al., *Fatal myocarditis and rhabdomyositis in a patient with stage IV melanoma treated with combined ipilimumab and nivolumab.* Curr Oncol, 2019. **26**(3): p. e418-e421.
4. Yamaguchi, S., et al., *Late-Onset Fulminant Myocarditis With Immune Checkpoint Inhibitor Nivolumab.* Can J Cardiol, 2018. **34**(6): p. 812.e1-812.e3.
5. Geisler, B.P., et al., *Apical ballooning and cardiomyopathy in a melanoma patient treated with ipilimumab: a case of takotsubo-like syndrome.* J Immunother Cancer, 2015. **3**: p. 4.
6. Yun, S., et al., *Late onset ipilimumab-induced pericarditis and pericardial effusion: a rare but life threatening complication.* Case Rep Oncol Med, 2015. **2015**: p. 794842.
7. Laubli, H., et al., *Acute heart failure due to autoimmune myocarditis under pembrolizumab treatment for metastatic melanoma.* J Immunother Cancer, 2015. **3**: p. 11.
